# Recent advances in understanding and managing Crohn’s disease

**DOI:** 10.12688/f1000research.9890.1

**Published:** 2016-12-21

**Authors:** Eduard F. Stange, Jan Wehkamp

**Affiliations:** 1 Department of Internal Medicine I, Medical University of Tübingen, Tübingen, Germany

**Keywords:** inflammatory bowel disease, Crohn's disease, IBD treatment

## Abstract

There is consensus that inflammatory bowel diseases (IBDs) are the result of “dysregulated” immune reactivity towards commensal microorganisms in the intestine. This gut microbiome is clearly altered in IBD, but its primary or secondary role is still debated. The focus has shifted from adaptive to innate immunity, with its multitude of receptor molecules (Toll-like and NOD receptors) and antibacterial effector molecules (defensins, cathelicidin, and others). The latter appear to be at least partly deficient at different intestinal locations. Host genetics also support the notion that microbe–host interaction at the mucosa is the prime site of pathogenesis. In contrast, even the latest therapeutic antibodies are directed against secondary targets like cytokines and integrins identified decades ago. These so-called “biologicals” have disappointing long-term results, with the majority of patients not achieving remission in the long run. A promising approach is the development of novel drugs like defensin-derived molecules that substitute for the missing endogenous antibacterials.

## Introduction

**Figure 1.**
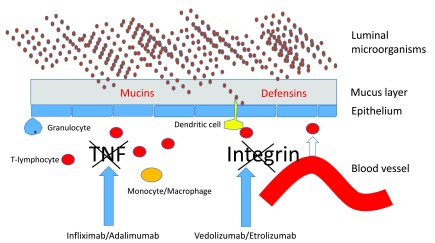
The principal barrier defects of mucins in ulcerative colitis and of defensins in Crohn’s disease as opposed to the prime therapeutic targets tumor necrosis factor (TNF) and integrins.

Crohn’s disease is a sometimes-devastating transmural inflammation that in principle may attack any site along the whole gut from mouth to anus. The last few years have seen some progress in the field of inflammatory bowel diseases (IBDs), where Crohn’s disease is one of the major two, besides ulcerative colitis. We understand better and we manage better, but we are far from curing these diseases. In many cases, we even fail to achieve remission, steroid-free long-term remission in particular. Why is this so? The main reason, in our view, is the major rift between the evolving barrier-centered concepts of pathophysiology and current conservative management. The latter is still based on drugs aimed at targets that were identified decades ago (like tumor necrosis factor [TNF] or integrins). It has become clear that the “dysregulated” adaptive immune response is not directed against gut tissue but against the intestinal microbiota ^1^. The primary defect obviously does not lie in the TNF or integrin system but in an imbalance of the gut microbiota and the defending mucosal barrier. In this brief review, we will outline this somewhat schizophrenic situation and consider possible solutions to this conundrum. A schematic representation is given in Figure 1 and discussed below.

## Understanding Crohn’s disease

### Microbiome

A seminal finding concerning the role of intestinal bacteria in Crohn’s disease was the observation of mucosa-attached and sometimes mucosa-invading bugs in Crohn’s disease ^2^. The fundamental relevance of this observation was rapidly accepted because, in nearly every IBD mouse model, ileitis or colitis were absent in the germ-free animal. It was also described that the microbial diversity was reduced and the composition of intestinal microbiota was altered in these diseases ^3^. The species of adherent–invasive *Escherichia coli* ^4^ was not specific for Crohn’s disease but appeared to be overrepresented, whereas the anti-inflammatory *Faecalibacterium prausnitzii* was underrepresented ^5^. In a recent meta-analysis, it was consistently demonstrated that *Clostridium coccoides* and *Clostridium leptum* counts were also low, as were those of *Bifidobacterium* ^6^. Nevertheless, there is no “helicobacter” in Crohn’s disease: it is not a simple infection like ulcer disease.

However, there was a significant difference between microbiota analyzed during inflammation and in its absence. This also holds at the mucosal level, where the systematic analysis of the microbiome was tremendously affected by the presence of inflammation ^7^. Therefore, the hen and egg question is still unresolved: we simply do not know which part of the alterations is secondary to inflammation (from other causes) or is indeed a primary pathogenic event ^8^. Nevertheless, the major role of the resident and usually symbiotic microbiota as a trigger and target of the immune response in IBD is undisputed.

### Barrier

In the old days, IBDs were considered to be autoimmune diseases, but evidence for autoantibodies is actually scarce in Crohn’s disease ^9^ and the typical anti-neutrophil cytoplasmic antibodies (ANCAs) in ulcerative colitis are probably secondary to cross-reactive microbial structures. Similarly, it has been difficult to pin down a defined T-cell mechanism leading to the massive T-cell response in the inflamed Crohn’s mucosa. If indeed a T-cell defect was operative, the basic restriction of the inflammation to the intestine would be hard to explain: extra-intestinal manifestations are the exception rather than the rule. Rather, the long-neglected barrier function of the gut mucosa has come into focus.

Initially, the first studies on barrier function demonstrated increased permeability to small molecules like lactulose rather than bacteria, allowing the prediction of imminent relapse during phases of remission ^10^. To understand the antibacterial barrier defects in the mucosa, its complicated multilayer structure is the key. The first line of defense is the mucus, consisting mostly of many different negatively charged mucins varying in size and carbohydrate content ^11^. Mucus also contains phospholipids, IgG and IgA antibodies, and, most importantly, positively charged antibacterial peptides. The relevant epithelial-derived antibacterials again form a multitude of vastly different compounds like defensins, cathelicidin, phospholipase A2, lysozyme, histones, and many others ^12^. Strikingly, and probably because of the enrichment of these natural endogenous antibiotics in the lower stratum of the mucus layer, the area immediately above the epithelium measuring approximately 100 μm is virtually sterile ^11^. Signaling occurs through epithelial membrane-bound Toll-like and intracellular NOD receptors recognizing a whole array of different bacterial-derived compounds like flagellin or muramyl dipeptide. This defense is indeed quite an achievement when the massive load of the microbiota in the lower intestine and colon lumen is considered: we are outnumbered by a ratio of 10 ^14^ to 1. Not surprisingly, these high bacteria count locations represent the main sites of Crohn’s disease.

The professional protective cells in the small intestine are the Paneth cells residing at the bottom of the crypts producing the α-defensins HD-5 and HD-6. HD-5 is a classical peptide antibiotic, whereas HD-6 forms nets in the crypt to restrict bacterial mobility ^13^. In ileal Crohn’s disease, Paneth cell function ^14^ and structure ^15^ are clearly compromised. In support of Paneth cells’ important role, a genetic defect in mouse Paneth cells is associated with inflammation. Of note, defective Paneth cell function may be corrected by Wnt signaling from monocytes, while Crohn’s disease monocytes lack this effect ^16^. In the colon principally all epithelial cells appear to be equipped for β-defensin production and indeed defects in the constitutive (HBD-1) and inducible (HBD-2 and HBD-3) defensin systems have been described ^17, 18^. Accordingly, the killing activity of mucosal extracts is diminished, whereas in ulcerative colitis it is even enhanced ^19^. Our data-based hypothesis of separate α- and/or β-defensin deficiencies ^20^ is attractive because it underscores and explains the occurrence of stable disease locations of ileal (so called “Paneth’s disease”) ^21^ or colonic Crohn’s, or the ileocolonic combination thereof. In contrast to Crohn’s disease, current evidence suggests that the lacking mucus or structurally defective mucins form the backbone of pathogenesis in ulcerative colitis ^22^.

The second layer of mucosal protection is formed by the continuous epithelium, stabilized by tight junctions that may also contribute to a “leaky barrier” during inflammation. In an ulcer, by definition, the epithelium is completely denuded and easily permeable to bacteria. Finally, if bacteria have gained access to the submucosa, mobile inflammatory actors like T-cells, dendritic cells, granulocytes, and monocytes/macrophages come into play. However, at this stage of inflammation, collateral damage to the tissue is unavoidable and sometimes irreversible.

### Host genetics

Genetics account for roughly half the risk of developing Crohn’s disease, the remainder being environmental factors like smoking, childhood hygiene, and early antibiotic use. Following the revolutionary observation of a genetic link of the intracellular bacterial receptor NOD2 ^23, 24^ with the risk of Crohn’s disease, multiple other sites in the genome were identified. The major ones are ATG16L1 as part of the autophagy machinery and IL23 receptor ^25, 26^. Other single nucleotide polymorphisms (SNPs) have suggested the endosomal stress response and the Wnt system as additional players, most of the above links (except IL23 receptor) hitting the Paneth cell ^21^. It must be noted that the total number of genetic links now exceeds 163 ^27^, but the majority has exceedingly low odds ratios that were statistically significant but actually irrelevant. Accordingly, only a small fraction of the total genetic risk has indeed been covered by this enormous number of linked SNPs. Nevertheless, these in-depth genome-wide investigations have identified the barrier ^27^ as well as host–microbe interactions as the “genetic architecture” ^28^.

In the context of disease location, a recent study has clearly underlined the notion that there is not just one homogeneous Crohn’s disease ^29^. Rather, even genetically ileal and colonic Crohn’s disease are different diseases, although they partly overlap. Taken together, the huge effort invested into genetics has paid off in helping to zoom in on the culprit, which is the lost war between microbiome and barrier. On the other hand, extensive data mining also may be misleading by ending up in an extremely complex map of a vast array of marginally associated mechanisms ^7^. Thus, the omics approach may, in the end, defocus the identification of the really pathophysiologically relevant disease events.

## Managing Crohn’s disease

### Anti-TNF and anti-IL12/23 antibodies

The advent of the anti-TNF antibodies was universally cheered in the field as a promising new approach sparing steroids and possibly substituting the aging immunosuppressants like azathioprine and methotrexate. Professional marketing efforts and support from many opinion leaders were successful in making the step from a very low-budget medication of oral immunosuppressants to a billion-dollar market. Adalimumab sales are in the absolute top level of the pharmaceutical drug world.

For many patients refractory to standard medications, the antibodies resulted in a dramatic improvement of their quality of life. Nevertheless, it should be noted that several conspicuous changes in performing, analyzing, and presenting Crohn’s disease studies were associated with this new drug entry ^30, 31^. First, the endpoint was softened from remission to “response”, defined as a mere drop of the CDAI (Crohn’s disease activity index) by 70–100 points. This improvement may be marginal to the patient if he/she is severely ill with a CDAI >300. Next, the percentage of patients in “remission”, the hard endpoint, was normalized not to the initial starter population but only to the subcohort achieving response early during the study: 39% of the 58% responders are only 23% of the initial population, for example ^30^. Thus, if referred to the initial population and followed for approximately 1 year, indeed >75% of those recruited in the trial do not achieve response or lose their response or remission despite continued treatment.

As far as side effects are concerned, we have now immunized a substantial part of the Crohn’s population to infliximab. Anti-drug antibodies are associated with allergic reactions but also loss of response, necessitating a switch to another antibody. Anti-TNF agents are associated with opportunistic infections ^32^, which are sometimes lethal. Other adverse events include psoriasis and probably melanoma. Anti-TNFs, however, have been cleared from causing lymphoma, but, if they are combined with azathioprine for suppressing antibody formation, non-Hodgkin or hepatosplenic T-cell lymphoma is still an issue ^33^. Thus, anti-TNF antibodies, although proven effective, have a relevant downside and should be used only if really indicated. The same holds true for ustekinumab ^34^, an IL12/23 antibody. It has recently been approved and will enter the market, but its limitations are very similar to those of the anti-TNF agents.

### Anti-integrin antibodies

Integrins promote the invasion of inflammatory cells into the tissue. The first anti-integrin antibody, natalizumab, was rapidly withdrawn from the market following the devastating occurrence of cerebral JC-virus infections. The more intestine-specific vedolizumab, directed against α4β7-integrin, is apparently safe in this regard and generally low in side effects ^35^. Similar to anti-TNF antibodies, it is active in both Crohn’s disease and ulcerative colitis but seems to take much longer to induce remission (again in the range of <20% over 1 year). It is also effective in patients refractory to anti-TNF antibodies, but here the remission rates are even lower ^36^. Novel anti-integrins like etrolizumab are promising but have not yet been proven effective in Crohn’s disease.

### Microbiome modifiers

Generally, antibiotics may offer some relief in this situation but have been disappointing. Metronidazole was shown to be effective in Crohn’s disease ^37^ but is associated with significant side effects. Ciprofloxacin is used especially for fistula patients ^38^, and, although there is only one study in combination with adalimumab, it appears to be beneficial ^39^. More promising is the non-absorbable antibiotic rifaximin, which was beneficial in an appropriately controlled trial ^40^. Due to the impossibility of eliminating the intestinal microbiota in the long term and the likely provocation of bacterial resistance, a continuous antibiosis with synthetic antibiotics is no promising option.

A more aggressive approach to modifying the microbiome is the transfer of “healthy” feces (the term “stool transplantation” should be avoided). Fecal transfer has given mixed results overall and even opposite results in two controlled trials in ulcerative colitis, one positive and one negative ^41^. The positive results were based on a special stool donor, which appeared to produce a remedy microbiome. Since the bacterial composition required to achieve remission is still not defined, further detailed analytical work in this area is urgently required. We still have to understand what the mechanisms and beneficial components of stool transfer are. Experience in Crohn’s disease is very limited and uncontrolled and should be critically evaluated.

### Pro-barrier agents

Probiotics have been suggested to modulate disease activity for a long time, but evidence for a benefit is largely restricted to ulcerative colitis. *E. coli Nissle* from the stool of a German soldier in World War I, in particular, has been shown to prevent relapse similar to mesalazine and may be used in those intolerant to this drug ^42^. Similarly, an Italian preparation (VSL3 #3) has recently been tested in Crohn’s disease and had no significant impact on clinical endpoints ^43^. Crohn’s disease seems to be more resistant to probiotics, which may be due to problems with one of their mechanisms of action, i.e. induction of defensins ^44^.

Another pro-barrier agent that is much more promising in ulcerative colitis compared to Crohn’s disease is lecithin. When given as a galenic ileal release formulation, lecithin has proven to be superior to placebo in acute ulcerative colitis ^45^ in a phase II trial. The phase III trial has been stopped owing to lack of efficacy.

Finally, the oral administration of defensin peptides may constitute a promising approach because it is directed towards a likely disease mechanism. Special modifications could be used to enrich the peptides further in the mucus, their natural “habitat”, and prevent epithelial attack of commensals. It is possible that this approach may also modify the microbiome, since, in animals genetically modified in their defensin system, microbial composition in the intestine was clearly altered ^46^. Defensins are currently in development for this clinical use but are still at an early stage. Alternatively, it may be possible, although far-fetched, to modulate crypt stem cell differentiation towards protective cells like Paneth and goblet cells ^47^ or even to transplant intestinal stem cells, since bone marrow transplants have been disappointing ^48^.

## Summary and outlook

The current situation is far from being satisfactory for Crohn’s disease patients, although treatment has improved with the advent of biologicals. Even with maximal therapy, the majority still does not achieve long-term remission and surgery rates have not gone down. The obvious remedy would be, of course, to develop a causal therapy directed at supporting the barrier against this constant natural microbial challenge. This is not an easy task, but if the efforts of both academics and the pharmaceutical industry are better focused on these issues, the outlook may be optimistic.

## Abbreviations

CDAI, Crohn’s disease activity index; IBD, inflammatory bowel disease; SNP, single nucleotide polymorphism; TNF, tumor necrosis factor.
